# Faba Bean: An Untapped Source of Quality Plant Proteins and Bioactives

**DOI:** 10.3390/nu14081541

**Published:** 2022-04-07

**Authors:** Delphine Martineau-Côté, Allaoua Achouri, Salwa Karboune, Lamia L’Hocine

**Affiliations:** 1 Agriculture and Agri-Food Canada, Saint-Hyacinthe Research and Development Centre, Saint-Hyacinthe, QC J2S 8E3, Canada; delphine.martineau-cote@agr.gc.ca (D.M.-C.); allaoua.achouri@agr.gc.ca (A.A.); 2 Department of Food Science and Agricultural Chemistry, Macdonald Campus, McGill University, 21111 Lakeshore, Ste Anne de Bellevue, QC H9X 3V9, Canada; salwa.karboune@mcgill.ca

**Keywords:** faba bean, pulse protein, nutritional quality, protein digestibility, DIAAS, PDCAAS, bioactive peptide, functional food

## Abstract

Faba beans are emerging as sustainable quality plant protein sources, with the potential to help meet the growing global demand for more nutritious and healthy foods. The faba bean, in addition to its high protein content and well-balanced amino acid profile, contains bioactive constituents with health-enhancing properties, including bioactive peptides, phenolic compounds, GABA, and L-DOPA. Faba bean peptides released after gastrointestinal digestion have shown antioxidant, antidiabetic, antihypertensive, cholesterol-lowering, and anti-inflammatory effects, indicating a strong potential for this legume crop to be used as a functional food to help face the increasing incidences of non-communicable diseases. This paper provides a comprehensive review of the current body of knowledge on the nutritional and biofunctional qualities of faba beans, with a particular focus on protein-derived bioactive peptides and how they are affected by food processing. It further covers the adverse health effects of faba beans associated with the presence of anti-nutrients and potential allergens, and it outlines research gaps and needs.

## 1. Introduction

Agricultural practices and dietary habits are subject to changes due to the global population increase and climate change. Current predictions suggest that the world population will reach 10 billion people by 2050 and that agriculture will be responsible for up to 30% of greenhouse gas emissions [1]. Livestock are significant contributors to these emissions by consuming substantial amounts of water and feed and by occupying large land surfaces [2]. Furthermore, excessive animal-based protein consumption is scientifically proven to be associated with various non-communicable diseases and metabolic disorders, such as obesity [3], type II diabetes [4], heart diseases [5], and cancers [6,7]. For the above reasons, along with increased consumer awareness about carbon emission reductions, there is a need to develop new, high-quality, and more sustainable protein sources. In this regard, legumes are well-acknowledged as valuable alternatives to animal-based proteins due to their economical, environmental, and medicinal properties [8]. The legume-based protein market is mainly composed of soy (*Glycine max* L.) and pea (*Pisum sativum* L.) [9]. However, as the demand for these products continues to increase, it is necessary to diversify and put forward new sources, such as from other pulse crops.

Pulses, such as lentils, chickpeas, and beans, are of high interest due to their well-established nutritional, economical, and environmental benefits [8]. Among pulses, the faba bean (*Vicia faba* L.), with its high protein content and agronomic advantages, represents an excellent, yet untapped, source of sustainable and quality dietary proteins. The faba bean is an annual dicotyledonous pulse (*Fabaceae* or *Leguminosae*) nut that has been grown for millennia in Asia, Africa, and in the Mediterranean region [10], but little is known about it/it remains underutilized in western countries [11]. It is well adapted to various climates, including boreal-types [12], and can, therefore, easily grow in colder regions, including in Canada [13]. Worldwide production of faba beans (5.7 million tonnes) is currently modest when compared to soy (353 million tonnes) and pea (14.6 million tonnes) [11]. The faba bean has agronomical, nutritional, and health benefits that may incite production growth in the future. Indeed, the faba bean is recognized as a pulse with a high ability to fix atmospheric nitrogen [14]. This characteristic can be wisely used to reduce nitrogen fertilizer applications that lead to detrimental ecological effects, such as eutrophication [15]. Therefore, the faba bean can be used in land rotation [16], in intercropping (to enrich soil) [17], and to increase the yield of other plants, such as barley [18] and wheat [19,20]. Faba bean intercropping also increases genetic diversity, which has a protective effect against the spread of diseases, as stated by a recent meta-analysis [21].

The faba bean, in addition to being a highly nutritive pulse, is not genetically modified (non-GMO) and not a regulated allergen—these are competitive advantages when compared to soy [22,23]. Although GMOs are safe to eat, some consumers are still reticent to include them in their diets [24]. The dry seed is mainly composed of starch, proteins, and dietary fibres (Table 1). The faba bean has a higher protein content than most pulses, including peas, chickpeas, lentils, and beans [25,26]. It is a rich source of vitamins and minerals and is low in fat. Similar to other pulses, it contains anti-nutritional factors, such as tannins, phytic acid, digestive enzyme inhibitors, oxalate, and lectins that can decrease the bioavailability and uptake of proteins and minerals during digestion and induce toxic effects [27]. One particularity of the faba bean involves the presence of vicine and convicine [28,29], which are associated with favism and are significant obstacles to faba bean utilization [30,31]. However, those undesirable compounds can be considerably reduced through food processing [28,29] and breeding strategies [32]. Newly improved faba bean varieties have been developed in recent years to further increase the nutritional qualities of the faba bean. Low tannins [33], as well as low-vicine and convicine [32] cultivars, are now available.

The faba bean, beyond its nutritional value, is also a rich source of bioactive compounds that have reported health-enhancing properties. These include phenolic compounds [68], resistant starch [69], dietary fibres [70], non-protein amino acids (L-DOPA [71], GABA [72]), and, foremost, bioactive peptides [73,74]. The faba bean, due to its richness in health-promoting constituents, has high potential in the development of new nutraceuticals and biofunctional food ingredients.

**Figure 1:**
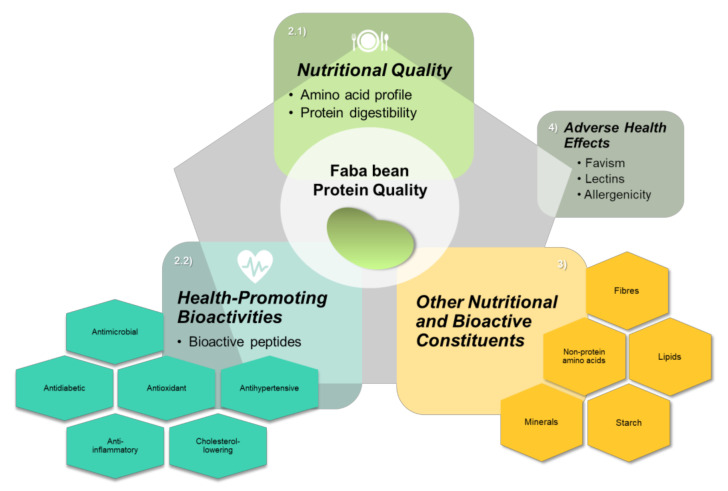
Faba bean protein quality is influenced by several factors, including nutritional qualities (amino acid profile and digestibility), health-promoting bioactivities, and other matrix constituents that can have beneficial (as well as adverse) nutritional and bioactive effects.

Many recent studies have focused on the nutritional and bioactive properties of faba beans [73,74,75,76,77]. The aim of this review is, therefore, to summarize the latest key findings related to the nutritional qualities of faba beans, as well as the biofunctional and health-beneficial properties, with particular focus on those related to proteins (Figure 1).

## 2. Faba Bean Proteins

The faba bean, as presented in Table 1, is a high protein pulse, with a higher protein content than peas and most pulses on the market, such as beans (22.17%), lentils (22.15%), and chickpeas (19.53%) [26], but lower than soy and lupin (40.0%) [25]. However, faba bean protein content is highly variable due to the substantial genetic diversity that exists among the species [41,42,75,78,79]. Indeed, several varieties have been developed via breeding over the years, depending on the consumer preferences in the region of origin, disease resistance, and the target market [80]. Faba bean varieties are therefore highly heterogeneous in size (less than 0.3 g to more than 1 g per seed) [71], color, and shape [80,81], depending on the cultivating area and the environmental conditions during the cropping year [82]. Soil composition, atmospheric nitrogen fixation efficiency, and environmental stress, such as drought, are all factors that can affect faba bean protein content [83,84]. Hood-Niefer, Warkentin, Chibbar, Vandenberg, and Tyler [42] have demonstrated that the protein content of 11 faba bean varieties grown in three western Canadian regions during two different years (2006 and 2007) fluctuated from 27.5 to 32.4%.

Most faba bean proteins are globulin-types, which is common to most pulses [51]. Plant proteins are classified into four main families, according to their solubilities in different solvents. Globulins are soluble in low-salt solutions, albumins in water, prolamins in 70% alcohol, and glutelins in alkaline solutions [85]. Globulin fractions count for 69.5 to 78.1% of total faba bean proteins, followed by glutelins (12.0 to 18.4%), prolamins (1.83 to 3.57%), and albumins (1.41 to 3.01%) [86]. The distributions of these proteins fractions depend on the cultivars and the environmental conditions [86].

Faba bean globulins are classified according to their sedimentation coefficients, into two major types, namely legumins (11S) and vicilins (7S) [87]. Legumins are the most abundant globulins in faba beans, composing up to 55% of the seed proteins [88]. They have hexameric structures, and each subunit is composed of acidic (α) and basic (β) peptide side chains that are retained together by a disulfide bond [88]. Vicilins have trimeric structures; each subunit is non-identical [88]. They are glycosylated and cysteine-free [87] and, therefore, are unable to form disulfide bonds. The legumins to vicilins ratio is an important factor in faba bean protein characterization, having an important impact on the functional properties of proteins [89,90]. The legumin to vicilin ratio is affected by numerous factors, including cultivars, environmental conditions, and processing conditions. Warsame et al. [91] found that the legumin to vicilin ratio ranged from 1 to 3 in 35 faba bean cultivars from across the world. Singhal, Stone, Vandenberg, Tyler, and Nickerson [89] reported that the legumin to vicilin ration ranged from 3.4 to 4.6 in the faba bean flour of seven Canadians cultivars cultivated in four different regions [89], but the difference was not significant. That same ratio was reported to vary from 3.76 to 5.40 in faba bean protein concentrates obtained by the air classifications of five Canadian cultivars cultivated in two different regions [90].

Faba bean legumins are more thermally stable than vicilins; their denaturing temperatures were reported to be 95.4 and 83.8 °C, respectively, for a protein concentration of 0.5 mg/mL [92]. Interestingly, denaturing temperatures of faba bean globulins were reported to be higher than pea and soy globulins [92]. Faba bean storage proteins have been studied recently with modern proteomic technics, and sixteen main storage proteins have been identified among six protein subunits of 97, 96, 64, 47, 38, and 32 kDa, with isoelectric points varying from 4.90 to 9.55 [93]. Among them, globulins were the most abundant proteins in the 64, 42, 47, and 38 kDa subunits [93].

Faba bean proteins have several advantages regarding the techno–functional properties of food formulation. Such applications have been recently reviewed elsewhere [94,95].

### 2.1. Nutritional Quality

The nutritional qualities of protein are determined by their amino acid compositions and their respective digestibility and bioavailability during the digestion process, fulfilling the dietary needs [96]. Numerous methods have been used to quantify the nutritional qualities of faba beans. These methods are briefly described in the next section.

#### 2.1.1. Assessment of Protein Nutritional Quality

Various metrics are used by regulatory agencies to rate protein sources and to regulate the protein quality claims of food products. Those metrics combine information relative to amino acid composition and protein bioavailability to determine global protein quality. In Canada, the protein efficiency ratio (PER) is used, which is a measure of metabolizable protein efficacy [97]. It is determined by a 28-day rat bioassay and consists of the ratio between weight gain and protein intake. The value is then normalized to a control to allow a study-to-study comparison (casein PER value is fixed to 2.5) [98]. The Food and Drug Administration (FDA) uses the Protein Digestibility Corrected Amino Acid Score (PDCAAS), which is a ratio between the first essential limiting amino acid amount for a given food commodity and that same amino acid amount in a reference protein (amino acid score) [99]. The ratio is then corrected according to the total fecal protein digestibility. The maximum PDCAAS value is 1; values above are truncated [99]. The PDCAAS has been subsequently criticized because it does not consider the digestibility of each amino acid individually [100]. Total fecal protein digestibility is also not entirely accurate since amino acids and short peptide absorption ends in the ileum, and unabsorbed proteins are further degraded in the large intestine by the microbiome [101]. Moreover, the truncated value does not provide enough information to evaluate the protein quality of a mixed diet [100] and to efficiently blend various protein sources in food applications to optimize amino acid composition [99]. The FAO has accordingly developed a new protein quality score based on these preoccupations, called the digestible indispensable amino acid score (DIAAS) [100]. This indicator is not truncated and is calculated for each essential amino acid to consider their respective ileal digestibility compared to a reference protein. The suggested reference protein varies according to the age group studied (newborn, children, and adults) because the amino acid requirement is not the same during those different stages of life [100]. The lowest DIAAS value among all essential amino acids of a specific food commodity corresponds to the global DIAAS value. DIASS values of 1 and above correspond to a high-quality protein and values between 0.75 and 0.99 correspond to a good-quality protein [100]. The total fecal protein digestibility for PDCAAS and the ileal-amino acid digestibility for DIAAS are preferably assessed in humans or animal models (pigs and rats) [100]. However, those assays are costly, invasive, ethically questionable, and time-consuming [102]. In vitro digestion assays are therefore widely used in the scientific literature to estimate protein digestibility. Strong correlations between protein digestibility data obtained in vivo and in vitro have been observed, which suggests the relevance of those digestion models [103]. The above indicators have been used in the literature to quantify faba bean protein nutritional quality; results are discussed in the sections below.

#### 2.1.2. Amino Acid Profile

The faba bean has an overall well-balanced amino acid profile that is similar to pea and soy (Table 2), containing a high amount of lysine, leucine, isoleucine, threonine, histidine, and aromatic amino acids [104,105]. However, sulfur-containing amino acids (methionine and cysteine) and tryptophan are present in lower amounts than soy [105]. This deficiency is common to most pulses [51] and is explained by the low content of those three amino acids in globulins [106]. Environmental conditions during cultivation have a significant impact on the faba bean amino acid profile [82]. Interestingly, the faba bean amino acid profile is complementary to cereals that are deficient in lysine but contain high levels of methionine and cysteine [38]. It can be advantageous to blend faba beans with cereals in food product formulations to optimize the amino acid composition. There is growing interest for such applications in the scientific literature, e.g., Laleg et al. [107] fortified wheat pasta with 35% of faba bean flour to enhance the essential amino acid content. This way, lysine, threonine, and branched aliphatic amino acid (leucine, isoleucine, and valine) content increased by 97, 23, and 10%, respectively. Furthermore, the protein efficiency ratio (PER) increased two-fold compared to wheat pasta [107], which suggests that this combination is beneficial to increase protein quality. Coda, Varis, Verni, Rizzello, and Katina [72] incorporated 30% of faba bean flour in wheat bread, which significantly increased the chemical score of lysine, threonine, and methionine compared to the control (wheat bread). Thus, these applications have excellent potential in the development of new food products that fulfill the nutritional needs of consumers and in improving product tastes and protein quality.

#### 2.1.3. Protein Digestibility 

Protein digestibility is a central element in protein quality assessment, by stating to what extent proteins are available for absorption during the digestion process. Plant-based protein digestibility depends on many factors, such as anti-nutritional content, cell wall integrity, particle size, protein structures, and protein interactions with the food matrix [109]. Those elements are intrinsic to the specific food commodities but are also affected by food processing [75]. The effects of food processing and anti-nutritional content on protein digestibility and quality are discussed in the following sections.

Additionally, physiological factors, such as a consumer’s age, also have crucial impacts on protein digestibility. In that respect, faba bean protein digestibility was recently evaluated in the context of infant nutrition [110]. The newborn digestive system is immature and the digestive fluid composition, enzyme activities, and pH in the digestive compartments are different than in adults [111]. This might have a substantial impact on protein digestibility. Gilani and Sepehr [112] have demonstrated that autoclaved-faba bean seed digestibility diminishes from 82 to 77% when assessed in young rats (5 weeks) compared to older rats (20 months), suggesting a significant impact of the age. Roux, Chacon, Dupont, Jeantet, Deglaire, and Nau [110] have replaced milk proteins by 50% plant-based proteins, such as pea, potato, rice, and faba bean in infant formulas. An in-vitro digestion system that mimics the newborn physiology was used; the degree of hydrolysis and the amino acid bioaccessibility were assessed as indicators of protein digestibility. Amino acid bioaccessibility was defined as the percentage of free amino acids in the digestate compared to the total amino acids in the infant formula. Interestingly, the degree of hydrolysis and amino acid bioaccessibility of the infant formula enriched with either faba bean or pea proteins was not significantly different from the milk control. However, rice and potato protein enrichments significantly decreased protein digestibility, suggesting that pea and faba beans were good alternatives for such a formulation [110].

#### 2.1.4. Effect of Processing on Protein Digestibility and Quality

Various processing strategies have been employed in the literature to increase faba bean protein digestibility and quality through biochemical and thermal processes.

##### Thermal Treatments

Reported thermal treatments in the literature included domestic processes, such as boiling, baking, and roasting, as well as industrial processes, such as extrusion cooking. Nosworthy, Medina, Franczyk, Neufeld, Appah, Utioh, Frohlich, and House [75] evaluated faba bean protein digestibility in rats and in vitro following extrusion, boiling, and oven-baking. Total fecal digestibility in rats ranged from 87.60 to 88.63% while in vitro total digestibility ranged from 76.79 to 82.22%, depending on the process used [75]. Faba bean showed the highest protein digestibility compared to various bean types, in which protein digestibility ranged from 57.58 to 87.41%. The authors also used total fecal protein digestibility to calculate the DIAAS values instead of the ileal amino acid digestibility. Most amino acids had a DIAAS value above 0.75, which corresponds to a good quality protein source [100], except for the sulfur-containing amino acids (0.54, 0.59, and 0.61 for extruded, boiled, and oven-baked faba bean flour, respectively) and tryptophan (0.70) following boiling. Aromatic amino acids (phenylalanine and tyrosine) and histidine had a DIAAS value above 1.00 for each process studied. They also reported the PDCAAS (58, 54, and 66%) and the PER (0.45, 0.85, and 0.66) for extruded, boiled, and baked faba beans, respectively. Thermal treatments can be beneficial for protein digestibility through their detrimental effects on heat-labile anti-nutrients, such as digestive protease inhibitors (trypsin and chymotrypsin inhibitors) [28]. Moreover, heat treatments can modify the native structures of proteins, affecting their digestibility. Carbonaro, Grant, and Cappelloni [106] demonstrated that intestinal digestibility in rats of purified faba bean globulins was significantly lower (78.79%) for thermally treated globulins (20 min at 120 °C) compared to the native globulins (95.08%) as a result of protein secondary structure changes during heat treatment [26]. Indeed, it was shown in various pulses that a high β-sheet content correlated to a decrease in protein digestibility due to the hydrophobic nature [113]. The structural change depends on the temperature and the moisture used during thermal treatment. At 60% moisture and a temperature of 65 °C (annealing), faba bean protein digestibility increased from 76.23 to 82.43%, but decreased to 73.26% at 120 °C and 30% heat moisture [26]. Moreover, the α-helix and β-sheet ratios increased following annealing and decreased after heat moisture treatment.

##### Biochemical Processes

Faba bean protein digestibility could also be improved using biochemical processes, such as germination, fermentation, and enzymatic treatment. Those processes involved the use of various endogenous, microbial, or commercial proteases to partially degrade dietary proteins and facilitate their absorption during the digestion process [72,114]. For instance, an increase in free amino acids up to 10-fold was observed during a solid phase fermentation of faba bean seeds with *Rhizopus oligosporus* used for faba bean tempeh preparation [115].

Besides increasing protein content in bread by mixing 30% of faba bean to wheat flour, the addition of fermented faba bean flour significantly increased protein digestibility in bread (74%) compared to raw flour (64%) [72]. Similar results were obtained in gluten-free corn-based fermented bread enriched with 50% of faba bean flour, where the protein digestibility increased from 53.9 to 72.3% [116]. For wheat pasta enriched with fermented faba bean flour, the in vitro protein digestibility increased from 49.2 to 54.3% and from 73.8 to 76.4% with a faba bean flour addition of 10 and 30%, respectively [114].

Berrazaga et al. [117] prepared a hybrid-yogurt by mixing faba bean protein (47%) and milk protein (53%) that could compensate for faba bean essential amino acid deficiency while diminishing animal protein content. The protein gel was prepared by chemical acidification or fermentation and fed to rats to assess the incidence of the selected process on protein digestibility. Fermentation could increase total protein digestibility (+7%), growth rate (+35%), and PER ratio (1.6 times) compared to chemical acidification. Contrarily, the potential use of fermented chickpea and faba bean flour as food ingredients had no significant enhancement in protein digestibility during an in vitro digestion process [118]. Those results suggest that fermentation conditions and microorganisms used are important factors that may influence fermentation efficiency to maximize protein quality.

Germination was also used in an attempt to improve faba bean protein quality. Setia, Dai, Nickerson, Sopiwnyk, Malcolmson, and Ai [36] determined, in vitro, the PDCAAS of faba bean seeds that were raw (56.2%), soaked (52.9%), and germinated for 72 h (56.5%). Germination had no significant impact on protein quality. However, the first limiting amino acid was tryptophan for the raw and soaked seeds and surprisingly threonine for the germinated seeds. During germination, endogenous enzymes are activated to support plant growth, and storage proteins are used as energy supply and amino acid stock for enzyme synthesis, which may explain the amino acid profile variation [36].

#### 2.1.5. Effects of Anti-Nutritional Content on Protein Digestibility and Quality

The presence of anti-nutrients, such as tannins, phytic acid, and trypsin inhibitors, have demonstrated a negative impact on faba bean protein digestibility.

##### Tannins

Tannins were shown to bind proteins and form insoluble complexes, reducing faba bean protein digestibility and bioavailability [70,119,120]. Tannin extracts from faba bean seed coats were shown to have higher precipitation potential against faba bean 11S globulins, followed by 7S globulins and 2S albumins and tannin-mediated precipitation occurring over a wide range of pHs (3 to 8) [121]. The faba bean mainly contains condensed tannins (proanthocyanidins), which are flavan-3-ol polymers. Their amounts can vary from 1.9 mg/g [70] to 2586 mg/100 g catechin equivalents [122]. These important variations can be explained by the varietal differences, environmental conditions [13], and quantification methods. Hydrolysable tannins (sugar esterified with a phenolic acid) are also present in faba beans, but at negligible amounts [121] compared to condensed tannins (58 mg/100 g of tannic acid equivalent) [122]. The most efficient way to decrease tannin content in faba bean is through the physical separation of the hull and the cotyledon. Based on reported studies, dehulling removes most (between 59.2 and 92.3%) of the phenolic compounds, including tannins [123,124]. Other processing, such as soaking, germination, and pressure-cooking, also proved to be effective household strategies used to reduce the levels of polyphenols and tannins in pulse-based foods, thereby enhancing the bioavailability of pulse proteins [45,81,125,126].

To eliminate nutritional quality issues related to tannins without using food processing strategies, low tannin faba bean varieties have been developed through breeding. This genetic trait is coded by two recessive genes (*zt-1* and *zt-2*) [33]. These varieties can be easily distinguished from the wild type varieties by their smaller and lighter seeds and their white flowers. Condensed tannin content was shown to be 0.03 g/kg compared to 7.12 g/kg for a high tannin variety [120]. The feeding of low tannin faba beans in rats significantly increases the protein efficiency ratio compared to high tannins (2.33 vs. 2.08), which confirms the nutritional improvement of low tannin varieties [120].

Although tannins have detrimental effects on protein digestibility, numerous health benefits are related to them. Faba bean tannins, obtained by acetone extraction and gel filtration chromatography, have demonstrated higher antioxidant activities (free radical scavenging and iron-reducing capacity) than low molecular weight phenolic compounds [68]. Faba bean tannin extracts, when fed to rats, showed a significant increase in serum HDL cholesterol and a slight decrease in total and LDL cholesterol, suggesting an improvement of the lipid profile [127]. Hence, tannins remain interesting healthy compounds even though they are classified as anti-nutrients. The beneficial health-related properties of various faba bean polyphenols were also reviewed in the literature [128].

##### Phytic Acid

Phytic acid (inositol hexaphosphate) is the main phosphorous storage found in faba bean cotyledons [123] and has a detrimental impact on protein digestibility by forming complexes with proteins and binding minerals, such as calcium, which are essential for digestive protease activity [129]. Phytic acid levels in faba beans vary among genotypes and environmental conditions [13], and were reported to be higher than peas [104], lentils, and chickpeas, but similar to beans and soy [29]. The effects of soaking and thermal treatments on phytic acid levels in faba beans varied among studies, from negligible changes to significant reductions due to differences in soaking and thermal conditions used (duration, pH, and pre-treatment) [29,123,130]. In contrast, phytase hydrolysis reduced the phytic acid level in faba beans by 89% under optimized conditions [131] and was efficient at increasing protein solubility and free amino acid levels during an in-vitro digestion process, principally during the gastric phase [131]. Furthermore, germination and fermentation both had important reductions on phytic acid levels in faba beans and an increase in calcium availability through either endogenous or exogenous phytase actions, which can be beneficial to maximize digestive protease activities [130,132].

##### Trypsin Inhibitor

Trypsin inhibitors decrease protein digestibility by inhibiting digestive protease activity in the gastrointestinal tract. Trypsin and chymotrypsin inhibitor activities were reported to be lower in faba beans than soy, beans, and chickpeas, and cooking was shown to reduce their activity levels below the limit of quantification [28]. Extrusion-cooking was also shown to reduce trypsin inhibitor activity by 50% [133]. Trypsin inhibitors have been purified and characterized in faba beans. For instance, *Vicia faba* cv. *Giza 843* trypsin inhibitor (VFTI-G1) is a polypeptide of 15 kDa that has an inhibitory effect against both trypsin and chymotrypsin to a lower extent [134]. Those inhibitory activities were reduced at temperatures above 60 °C [134]. Nonetheless, faba bean trypsin inhibitors possess properties that make them appealing from a therapeutic point of view. VFTI-G1 (Bowman–Birk-type) revealed anticancer properties, emphasized through an anti-proliferative effect demonstrated on a hepatocellular carcinoma cell line (HepG2) [134]. Another trypsin and chymotrypsin inhibitor of 7.5 kDa (Bowman–Birk-type) from faba was shown to have fungicide properties [135].

### 2.2. Faba Bean Health-Promoting Bioactive Properties

Bioactive peptides are short peptides of 2 to 20 amino acids that are either naturally present in the food matrix or released through protein hydrolysis [136]. Those amino acid sequences have no specific activities while embedded in the initial protein structure, but become highly active after protein hydrolysis [136]. A broad range of bioactivities are attributed to peptides, such as antioxidant, antidiabetic, cholesterol-lowering, anti-inflammatory, anticancer, antihypertensive, opioid, and antimicrobial, among others [77,136,137]. The relationship between chemical structure and bioactivity is still under investigation and varies according to the specific bioactivity [138,139]. However, the peptide length, charge, amino acid composition, and particular order, as well as the hydrophobic amino acid ratio content, are all factors of significant influence for many bioactivities [74,140].

Bioactive peptides have been identified and isolated from a wide range of food commodities, including legumes that are considered significant sources [140]. Some recent studies suggest that faba bean proteins have great bioactive potential (Table 3). Moreover, León-Espinosa, Sánchez-Chino, Garduño-Siciliano, Álvarez-González, Dávila-Ortiz, Madrigal-Bujaidar, Téllez-Medina, and Jiménez-Martínez [77] conducted a bioinformatics analysis using the BIOPEP database [141] to investigate potential bioactivities in main faba bean storage proteins. The algorithm used computes known bioactive fragment frequencies in a given protein sequence. This analysis revealed a high occurrence of potential antihypertensive, antioxidant, and other various biological activities. Legumin A was shown to have the highest bioactive fragment frequency, followed by convicilin and vicilin [77]. However, the truly released fragments will highly depend on the enzymes used to hydrolyze the proteins and the hydrolysis efficiency, which need to be investigated through further analyses and experimental work.

#### 2.2.1. Faba Bean Gastrointestinal Hydrolysates with Potential Beneficial Health-Related Bioactivities

Some studies [73,74,76,77,144,145,146,147] have demonstrated that faba bean proteins hydrolyzed with gastrointestinal proteases have the potential to promote various beneficial health-related bioactivities that go beyond their nutritional properties. For now, most evidence concerning these health benefits are based on in vitro results. In this regard, Felix, Cermeño, and FitzGerald [145] studied the antioxidant, antihypertensive, and antidiabetic effects of a sunflower oil-based emulsion stabilized with a faba bean protein concentrate after an in vitro simulated gastrointestinal digestion with pepsin and Corolase PP^®^ (bacterial endopeptidase mix). The free radical scavenging properties of the emulsion were enhanced following digestion when assessed with the ferric reducing antioxidant power (FRAP) assay, possibly due to the release of small peptides. Meanwhile, the oxygen radical absorbance capacity (ORAC) value decreased after digestion, showing that the free radical scavenging mechanisms of those peptides were better highlighted with the FRAP assay. The antihypertensive and antidiabetic properties of the emulsion also increased following digestion [145]. Those properties were assessed through the angiotensin-converting enzyme (ACE) and dipeptidyl peptidase 4 (DPP-IV) inhibition assays. Following digestion, the enzyme inhibition activities of the emulsion increased from 23 to 60% for ACE and from 3 to 11% for DPP-IV [145]. Those results suggest that highly bioactive peptides are potentially released from faba bean proteins during gastrointestinal digestion. However, further investigation is needed since those peptides were neither purified nor specifically characterized.

León-Espinosa, Sánchez-Chino, Garduño-Siciliano, Álvarez-González, Dávila-Ortiz, Madrigal-Bujaidar, Téllez-Medina, and Jiménez-Martínez [77] also observed antioxidant properties (2,2′-diphenyl-1-picrylhydrazyl (DPPH) and 2,2′-azinobis-(3-ethylbenzothiazoline-6-sulfonate) (ABTS) scavenging activities) of a faba bean protein isolate digested with either trypsin, chymotrypsin, or pancreatin. It was demonstrated that faba bean protein hydrolysis with trypsin was more efficient at liberating antioxidant peptides than chymotrypsin and pancreatin, which could be explained by their respective cutting sites that generated different peptides. Indeed, Parya Samaei, Ghorbani, Tagliazucchi, Martini, Gotti, Themelis, Tesini, Gianotti, Gallina Toschi, and Babini [146] demonstrated that only 0.2% of the peptides identified were homologous when a faba bean protein digestate was prepared with either pepsin, trypsin, or alcalase. Moreover, when the enzymes were used in combination, only 26% of the peptides identified where homologous in the pepsin–trypsin and trypsin–pepsin digestates, which suggests the importance of the sequential enzyme order of addition. In that case, the usage of alcalase alone, and a combination of pepsin and trypsin, were the most efficient enzymes to maximize antioxidant activities. Moreover, numerous peptides with sequences or fragments homologous to antioxidant peptides already identified were found in those faba bean digestates.

It was also demonstrated that faba bean protein trypsin digestate had a protective effect against colon cancer in mice by reducing the number of preneoplastic lesions induced by either a high-fat diet or azoxymethane injections [77]. The lowest hydrolysate dose tested (10 mg/kg) was shown to have the highest effect. The same hydrolysate also caused a cholesterol-lowering effect, which was highlighted by the improvement of the lipidic profile. Dietary peptides can lower blood cholesterol by either decreasing exogenous cholesterol absorption in the intestine or in diminishing endogenous cholesterol biosynthesis. Ashraf, Awais, Liu, Khan, Tong, Ma, Wang, Zhou, and Zhou [147] demonstrated that faba bean peptides (<3 kDa) obtained from the hydrolysis of a thermally treated protein isolate with pepsin and trypsin decreased cholesterol solubility into micelles and inhibited the 3-hydroxy-3-methylglutaryl coenzyme A (HMG-CoA) reductase, an important enzyme of the mevalonate pathway responsible for cholesterol synthesis. Its inhibition promotes the use of blood LDL cholesterol instead of de novo cholesterol synthesis [147]. Those action mechanisms could explain the cholesterol-lowering effects of faba bean peptides observed in vivo by León-Espinosa, Sánchez-Chino, Garduño-Siciliano, Álvarez-González, Dávila-Ortiz, Madrigal-Bujaidar, Téllez-Medina, and Jiménez-Martínez [77].

Since fermentation is an effective process to enhance the bioactive properties of a food product, by releasing bioactive peptides through various proteolytic activities [148], Jakubczyk, Karaś, Złotek, Szymanowska, Baraniak, and Bochnak [73] recently studied the potential of fermented faba bean flour by *Lactobacillus plantarum* to produce bioactive peptides against metabolic syndrome during an in vitro digestion process that mimics human digestion. The digestion of the fermented flour was pursued by adding α-amylase, pepsin, and pancreatin subsequently. A peptide-rich fraction (peptide 9), as reported in Table 3, with antiradical activity (EC_50_ = 0.02 mg/mL) and inhibition potential against ACE (IC_50_ = 0.05 mg/mL), lipoxygenase (LOX) (IC_50_ = 0.10 mg/mL), and pancreatic lipase (IC_50_ = 0.46 mg/mL), was obtained. It is reported that inhibition of pancreatic lipase diminishes lipid absorption in the intestine and helps to restore calorie intake balance in patients with metabolic syndrome [149]. LOX is also known as an enzyme involved in the inflammatory response and chronic inflammation associated with various metabolic dysfunctions, including obesity and type II diabetes [150]. Nevertheless, the identified peptides were not tested individually, and their specific bioactivities remain to be confirmed.

In their study, Karkouch, Tabbene, Gharbi, Ben Mlouka, Elkahoui, Rihouey, Coquet, Cosette, Jouenne, and Limam [74] isolated five peptides (peptides 1 to 5 in Table 3) with either antioxidant, antityrosinase, or antibiofilm properties from faba bean proteins digested overnight with trypsin. One of these peptides was also isolated by Rizzello, Verni, Bordignon, Gramaglia, and Gobbetti [142] for its fungicide properties (peptide 3), revealing that bioactive peptides could have multifunctional properties [137]. Among the peptides isolated, peptide 4 has the highest DPPH free radical scavenging activity with an effective concentration (EC_50_) of 0.25 ± 0.02 mM. However, only peptide 3 has demonstrated a reducing capacity of Fe^3+^ to Fe^2+^ (EC_50_ = 0.31 ± 0.03 mM) and an iron-chelating activity (EC_50_ = 2.40 ± 0.31 mM). Interestingly, this peptide, with the highest hydrophobic amino acid residue ratio (56%), contributed to those properties [74]. Chelation of transition metal ions contributes to peptide antioxidant properties because they act as catalysts in some free radical formation reactions [151,152]. Tyrosinase is an enzyme involved in melanin biosynthesis and tyrosinase activity abnormalities are associated with skin pigmentation diseases and even skin cancer [74]. Peptide 4 has the highest tyrosinase inhibition capacity, with an IC_50_ of 0.14 ± 0.01 mM.

Other promising bioactivities were recently identified by Dugardin, Cudennec, Tourret, Caron, Guérin-Deremaux, Behra-Miellet, Lefranc-Millot, and Ravallec [76]. It was shown that faba bean protein gastrointestinal hydrolysate could play a role in regulation of food intake in modulating the secretion of incretins, such as glucagon-like peptide-1 (GLP-I) and cholecystokinin (CCK) in a dose-dependent manner in a murine intestinal cell model. Nonetheless, the effect was inferior when compared to pea, potato, oat, and wheat protein hydrolysate. Interactions among faba bean protein with opioid receptors were also demonstrated, but the binding capacity decreased significantly after gastrointestinal digestion. Moreover, it was not investigated whether the hydrolysate had the capacity to cross the intestinal barrier to induce this effect in vivo. Interactions with opioid receptors in the portal vein could induce satiety and play a role in food intake regulation. Anti-inflammatory activity was also investigated, but no effect was observed. The faba bean hydrolysate was shown to have greater in vitro antihypertensive, antidiabetic, and antioxidant activity, when compared to oat, wheat, potato, and pea gastrointestinal hydrolysate [76].

Cal et al. [153] have demonstrated that faba bean protein hydrolysate can play a beneficial role in skeletal muscle health in promoting skeletal protein synthesis and in preventing muscle loss caused by chronic inflammation. These activities could be beneficial to fight against sarcopenia during aging, among others. Hydrolysate of various plant-based proteins (chickpeas, soy, Asian rice, and spirulina) were prepared with a food grade endopeptidase. Interestingly, the faba bean hydrolysate was the only one with a protein synthesis promoting activity [153]. The activity of this novel functional ingredient was validated in vitro with cell models and in vivo in a pre-clinical study. The peptide profile of this complex functional food ingredient was screened for anti-inflammatory and protein synthesis promoting activity using a predictive machine learning approach [144]. Two peptides were discovered, HLPSYSPSPQ and TIKIPAGT with protein synthesis promoting and anti-inflammatory activity, respectively. Moreover, both peptides were resistant to gastrointestinal digestion, when administred in the complex ingredient forms. They were able to cross the intestinal barrier and were resistant to human sera peptidases [144], which demonstrates excellent in vivo bioactive potential.

Thus, the faba bean protein could be considered a promising source of bioactive peptides. However, studies remain limited, and many more bioactive peptides need to be elucidated. Moreover, peptides released in a context that mimics human digestion have been scarcely investigated to date. Further studies are therefore required to evaluate the health benefits of introducing faba bean protein in the diet. Studies using more physiologically relevant assays, such as cellular or animal models, are required to confirm the results obtained with biochemical tests. Moreover, bioavailability, uptake, stability, and resistance to brush border and serum peptidases are factors that need to be addressed to determine if those peptides can reach their targeted sites of action in a sufficient concentration to induce respective beneficial health-related effects. Peptide absorbability depends on many factors, including length, charge, and hydrophobicity, as well as the food matrix composition [154]. Fibre-rich matrices were shown to enhance bioactive peptide absorption and protect peptides against chemical degradation, whereas lipid-rich matrices substantially decreased peptide absorption [154]. According to these facts, the faba bean, with its very low lipid content and high fibres levels, could be an optimal matrix to facilitate dietary peptides bioavailability and absorption. Further investigations are needed to validate this hypothesis.

#### 2.2.2. Anti-Microbial Bioactivities of Faba Bean Enzymatic Hydrolysates

Faba bean protein hydrolysates have been produced in non-physiological conditions to create new biofunctional food ingredients. Rizzello, Verni, Bordignon, Gramaglia, and Gobbetti [142] have isolated two faba bean peptides with fungicide properties from a commercial pulse flour mix (pea, lentil, and faba bean) hydrolysate obtained by a protease mix commonly used in the baking industry. Those two peptides (peptides 6 and 7) as reported in Table 3 had a high hydrophobic residue ratio (36 and 50%) in common. The mixed flour hydrolysate was added to the baking products and increased shelf life without impairing the organoleptic properties. This is a promising finding considering that one of the main problems encountered with bioactive peptide enrichment in food products is the development of an undesirable bitter taste [155]. Karkouch, Tabbene, Gharbi, Ben Mlouka, Elkahoui, Rihouey, Coquet, Cosette, Jouenne, and Limam [74] also identified four peptides in a faba bean trypsin hydrolysate with antimicrobial activities. These peptides had the ability to block biofilm formation by *Pseudomonas aeruginosa*; for peptides 3 and 5 (Table 3), the effect was dose-dependent. Hydrophobic and basic residues seem to be important for this activity. Indeed, hydrophobic and basic amino acids can disrupt cell–cell interactions and, therefore, inhibit biofilm formation [74].Thus, faba bean protein hydrolysate could potentially be used as a biofunctional food ingredient acting as a natural preservative agent [142]. Similarly, a lactic acid bacteria (LAB) growth-promoting peptide was also obtained from faba bean proteins digested with alcalase [143]. This short peptide of only three amino acids (peptide 8), as reported in Table 3, could increase viable LAB count by one-log compared to the control, and act as a food ingredient to maintain the probiotic counts in various products [143].

## 3. Other Nutritional and Bioactive Constituents of Faba Beans

### 3.1. Starch

Carbohydrates, similar to other plants belonging to the *Leguminosae* family, are the major constituents of faba beans, where the starch component counts for approximately 40% of the whole seed [45]. Li, Yuan, Setia, Raja, Zhang, and Ai [69] reported that starch is conceptually characterized as rapidly digestible (if digested in less than 20 min), slow digestible (between 20 to 120 min), or resistant to digestion (more than 120 min). In the case of the raw faba bean, rapidly digestible starch was shown to account for 15.3%, slowly digestible for 34.5%, and resistant starch for 46.7%. Both slowly digestible and resistant starch help maintain the satiety feeling longer and contribute to a low glycemic index by flattening the blood glucose peak following food intake, thereby having a preventive effect against type II diabetes [156]. In addition, resistant starch has a prebiotic effect; it is undigested and unabsorbed in the small intestine but fermented in the large intestine by the microflora into short-chain fatty acids, inducing health benefits in the large intestine, such as reducing inflammation and preventing colon cancer [157]. Compared to cereals, the starch digestion rate in raw pulses is lower due to many factors, including starch granule morphology (large smooth rounded to oval shape granules [69]), crystallinity polymorph (C-type), and high amylose content, which reduces access to digestive enzymes [158,159].

There are about 20%–30% amylose (AM) and 70%–80% amylopectin (AP) in normal starch granules. Amylose is a linear glucose polymer (α-1,4 linkages), whereas amylopectin is a branched glucose polymer (α-1,4 and α-1,6 linkages). These two polymers can be combined into five different starch structural levels, including whole granule architecture (1–100 nm), growth rings (120–400 nm), blocklets (20–500 nm), amorphous and crystalline lamellae (9 nm), and AP and AM chains (0.1–1.0 nm) [160]. Physicochemical and structural properties of starches determine their applications in food and non-food industries. Amylose forms very compact structures; thus, it is hardly digestible [161]. Differences in the levels of amylose in faba beans, varying from 18.6 to 44.4% of total starch, could be explained mainly by the analysis method used (enzymatic or potentiometric approach) [36,69,158]. Their levels were similar to other legumes (lentils and peas) but higher than corn and tapioca [69].

Furthermore, the faba bean starch digestion rate is affected by cooking, reaching 88.1% digestibility within 20 min [69], similar to other legumes and cereals [158]. Enriching durum wheat pasta with 35% faba beans not only enhanced its protein and essential amino acid content and strengthened its protein network, but also resulted in a low glycemic and insulin index in healthy volunteer consumers [162]. Similarly, Tazrart et al. [163] demonstrated that the enrichment of fresh wheat pasta with faba bean flour decreased the pasta glycemic index in a dose-dependent manner. The glycemic index dropped from a value of 95.9 for the control to 91.9, 83.4, and 71.3 for pasta enriched with 10, 30, and 50% of faba bean flour, respectively. Moreover, the more faba bean flour added, the more resistant starch increased in the pasta, from 1.44 g/100 g to 1.86, 2.25, and 2.47 g/100 g, respectively. On the other hand, processing conditions for pasta preparation have a substantial impact on pasta structure and, thus, digestibility [162]. During cooking, starch undergoes gelatinization, which irreversibly disrupts the starch granule structure [164].

To increase native starch digestibility, various physical, chemical enzymatic, and biotechnological methods are applied. These techniques have been found to change the surface properties, polarity, and linearity of the molecular chains, the degree of substitution, the polymeric, granular, and crystalline structures, amylose to amylopectin ratio, solubility, viscosity, pasting, gelatinization, swelling, water absorption, and emulsifying properties of starch [165]. Using chemical modification through cross-linking reactions [158] showed that phosphorylation of gelatinized faba bean starch decreased the digestion rate slightly, but further optimization of the phosphorylation reaction is required to optimize the effects [158]. Another factor that can influence starch digestibility is the presence of α-amylase inhibitors. However, this activity was reported to be very low in faba beans (18.9 U/g in raw seeds compared to 248 U/g in raw kidney beans), highly heat-sensitive [124], or completely absent [123].

### 3.2. Fibres

The faba bean is a very important source of both soluble and insoluble dietary fibres (non-starch polysaccharides), as shown in Table 1. Faba bean insoluble dietary fibres are mainly composed of hemicellulose (8.92 g/100 g), cellulose (8.33 g/100 g), and lignin (2.00 g/100 g) [132]. Dietary fibre consumption is associated with many health benefits, including improvement of cholesterol profiles and preventive effects against diabetes, obesity (increasing satiety and maintaining it over time), and colon cancer [166]. The recommended daily fibre intake in Canada is 25 g for women and 38 g for men [167], but the actual fibre consumption is below those targets (19.1 g for men and 15.6 g for women) [168]. The use of faba bean flour to improve the nutritional and functional features in food-making would certainly increase a consumer’s recommended daily intake.

One of the health-promoting properties of fibre is related to its ability to bind bile acids during digestion and to decrease circulating LDL cholesterol. Bile acids are formed from cholesterol transformation in the liver and are indispensable to lipid digestion [169]. After digestion, they are usually reabsorbed in the ileum; however, their sequestration by fibre prevent their reabsorption and promote the use of more blood LDL cholesterol to produce new bile acids [169]. In this regard, Çalışkantürk Karataş, Günay, and Sayar [70] assessed whole faba bean and faba bean hull fraction capacities to bind bile acids during an in-vitro digestion process. Their results indicated that faba bean whole flour and the hull fraction had a bile acid-binding capacity of 14.6% and 282.6% (normalized to cholestyramine capacity), respectively. Interestingly, the bile acid-binding effect was attributed to tannin content rather than total fibre content [70].

Another health-promoting property of fibre involves its prebiotic effects. Fibre is resistant to gastrointestinal digestion but can be fermented into short-chain fatty acids in the large intestine by the microflora, which prevents the growth of undesirable bacteria and contributes toward preventing colon cancer [70,170]. Çalışkantürk Karataş, Günay, and Sayar [70] assessed the potential of faba bean gastrointestinal digestion residue to promote gut microbiota fermentation. It was shown that faba bean digest residue promotes the formation of short-chain fatty acids, mainly acetic acid (56.9 µmol/100 mg residue), butyric acid (36.1 µmol/100 mg residue), propionic acid (23.9 µmol/100 mg residue), and valeric acid (8.8 µmol/100 mg residue). A similar pattern was obtained by Gullón, Gullón, Tavaria, Vasconcelos, and Gomes [170], with the most abundant short-chain fatty acid being acetic acid, followed by butyric acid and propionic acid after 48 h of fermentation. Moreover, the faba bean gastrointestinal digest residue was shown to promote the growth of various healthy intestinal bacteria genera, such as Bifidobacterium, Lactobacillus, Enterococcus, Bacteroides, and Prevotella [170]. This growth-promoting effect can be attributed to dietary fibre and resistant starch, but also to α-galactosides that have well-established prebiotic effects. The faba bean was shown to contain an important amount of raffinose (4.8 g/kg), stachyose (10.1 g/kg), and verbascose (22.8 g/kg) [171], which contribute to the whole seed prebiotic effect.

### 3.3. Lipids

The faba bean has a very low lipid content (Table 1), similar to peas and other pulses, but considerably lower than soy. The fatty acid profiles are comparable for these legumes and are composed mainly of beneficial monounsaturated (oleic acid) and polyunsaturated (linoleic acid) fatty acids. These unsaturated fatty acids have demonstrated beneficial health-related effects, such as lipid profile improvement (an increase of sera HDL cholesterol) that contributes to heart disease prevention [172]. Nonetheless, the faba bean is a minimal lipid source, which can be advantageous in food applications. Indeed, pulse flours and pulse-derived food ingredients could develop bitter and beany off-flavours during storage [173], which is mainly attributed to lipid degradation by endogenous lipases and lipoxygenases [173]. Endogenous lipoxygenase activity, in addition to its low-fat content, was reported to be lower in faba beans than in peas and soy, which decreases the risks of these degradation reactions during storage [174].

### 3.4. Minerals

The faba bean, in addition to being an excellent source of protein and starch, contains valuable mineral micronutrients. More precisely, it is a rich source of potassium, iron, and zinc [104,175]. The faba bean contains a very low sodium amount [175], which is a desirable trait considering that high sodium consumption is associated with heart-disease preponderance [176]. The primary issue with plant-derived minerals involves their poor bioavailability during the digestion process [177] due to anti-nutrients, such as oxalic and phytic acid. Oxalic and phytic acid, with their multiple acidic functional groups, can bind minerals to form insoluble salts in the intestine (phytate and oxalate, respectively), which decrease the uptake of essential minerals [178]. Total oxalate content in the faba bean was shown to be 241.50 mg/100 g, which is similar to peas (244.65 mg/100 g) and lower than soy (370.49 mg/100 g) [29]. Oxalate content can be partially reduced through soaking and cooking [29,179]. Moreover, biochemical processes are suitable for decreasing phytic acid content in faba beans and increasing mineral bioavailability. For instance, germination significantly increased iron, copper, and calcium bioavailability, maintained that of manganese, but decreased zinc accessibility [180]. It was also shown that faba bean flour hydrolysis with phytase increases iron absorption in rats [181]. Among the other factors that influence plant-derived iron bioavailability is the binding to phytoferritin, an iron storage protein. Recent studies suggest that phytoferritin-bound iron is protected from anti-nutrient precipitation due to protein coating [182] and it is more easily absorbed in the intestine [183]. However, to exert this beneficial effect, phytoferritins have to resist the gastric digestion phase [183]. Phytoferritins are composed of two subunits (H-1 and H-2); the H-2 subunit was shown to be more stable and resistant to pepsin hydrolysis than H1. Interestingly, faba bean phytoferritins have a higher proportion of H-2 subunits (H-1:H-2 ratio of 1:6) compared to soy (1:2) and peas (1:1), which suggest that faba bean phytoferritins have the potential to resist gastric conditions [184], and thereby increase iron bioavailability. Further research is needed to confirm this hypothesis.

### 3.5. Non-Protein Amino Acids

The faba bean also contains a significant amount of non-protein amino acids that have beneficial health-related effects, particularly L-3,4-dihydroxyphenylalanine, also called levodopa or L-DOPA. L-DOPA, which is a dopamine precursor. Synthetic versions of this compound are widely used to improve motor functions in patients with Parkinson [185]. The synthetic version of L-DOPA causes many side effects, which explain the growing interest in finding natural sources [186]. It was demonstrated that the plasma levels of L-DOPA increased after the consumption of 250 g of cooked faba beans by healthy volunteers and patients with Parkinson’s [187]. The motor functions of patients with Parkinson’s improved up to 4 h after faba bean ingestion, an effect that was similar to a treatment composed of 125 mg of L-DOPA and 12.5 mg of carbidopa. These results suggest that a realistic serving of faba bean contains a sufficient amount of L-DOPA to induce a clinical effect [187]. L-DOPA is naturally formed from tyrosine in the faba bean and accumulates in various organs of the plant, including the leaves [188], flowers [189], and seeds, where it reaches its highest concentrations while the plant is still immature. Mature dried seeds still contain an important amount [71,188]. Purves, Zhang, Khazaei, and Vandenberg [71] quantified L-DOPA in 42 faba bean cultivars with high genetic diversity in terms of seed size, tannins, and vicine and convicine content, and the L-DOPA content ranged from 0.09 to 1.15 mg/g [71]. Other studies reported values in the same order of magnitude [190,191]. In addition to the cultivar, environmental stress and processing can interfere with the accumulation of this specific secondary metabolite [188]. L-DOPA was shown to be either completely or partially destroyed by thermal processes, such as boiling [188,191] and roasting [191]. On the contrary, Abdel-Sattar et al. [192] revealed that sprouting increased L-DOPA content of the faba bean. In their study, a methanolic extract of the faba bean was shown to have anti-Parkinson’s effects in a mice model; the effects drastically improved after germination, which coincided with an increase in flavonoids, phenolic acids, saponins, and aromatic amino acids [192].

The faba bean also contains γ-aminobutyric acid (GABA), which is an inhibitory neurotransmitter amino acid that has blood pressure-lowering effects [193]. It is a secondary metabolite formed from glutamic acid by glutamate decarboxylase, which accumulates in faba bean seeds due to environmental stress [194,195,196,197]. Germination [198] and fermentation [72,115,199] are both very useful processes to increase GABA in the faba bean. For instance, Coda, Varis, Verni, Rizzello, and Katina [72] fortified wheat bread with either 30% raw or 30% of fermented faba bean flour. The addition of faba bean flour to the dough caused an increase of GABA content compared to the wheat dough. Fermentation of faba bean flour further increased GABA content from 89 to 315 mg/kg of flour. Coda, Varis, Verni, Rizzello, and Katina [72] reported that the GABA content in a 50 g serving of bread containing fermented faba bean flour is a sufficient dose to trigger beneficial health effects.

## 4. Faba Bean Adverse Health Effects

### 4.1. Favism

Consumption of faba beans, despite the positive repercussions surrounding nutrition and the environment, might pose some health hazards to certain groups of consumers due to the presence of certain components. Faba beans contain pyrimidine glycosides vicine and convicine, which are precursors of the aglycones divicine and isouramil. These are the main factors of favism, a genetic condition that may lead to severe hemolysis after faba bean ingestion [200,201]. Favism is a hemolytic anemia that can be developed among people with a deficiency in glucose-6-phosphate dehydrogenase (G6PD). This enzyme deficiency affects around 330 million people worldwide, mainly in Africa, South America, the Mediterranean region, and South-East Asia [202]. It is a recessive X-linked trait; thus, predominantly affecting men [203]. Without G6PD, red blood cells are unable to re-establish the oxidative imbalance caused by isouramil and divicine, which leads to oxidative damage and hemolysis [201]. Ivarsson and Neil [41] analyzed 16 faba bean varieties, including low and high tannin varieties, and the values ranged from 6.64 to 7.90 g/kg and from 2.48 to 4.41 g/kg for vicine and convicine, respectively [41]. Low-vicine and convicine varieties (*vc*-) have been developed, and genetic markers have recently been identified to facilitate the distinction between the two genotypes [204]. The genetic improvements to reduce vicine and convicine in faba bean are reviewed elsewhere [32]. In low vicine and convicine varieties, the concentrations were reported to vary from 0.13 to 0.73 and 0.009 to 0.037 mg/g, respectively, a substantial decrease compared to standard varieties [71]. It was recently demonstrated that the consumption of 500 g of *vc*-faba bean seeds per 70 kg of body weight did not cause oxidative damage or hemolysis in G6PD deficient patients. Those results suggest that *vc*-faba bean varieties are potentially safe for patients who are lacking G6PD, but further studies with more patients are required [205]. Moreover, new analytical procedures have been developed to quantify vicine and convicine in food products to assure food safety [71,206,207].

### 4.2. Presence of Lectins

Faba beans also contain lectins, which are low molecular weight proteins (~18 kDa) [208] that have characteristic binding capacities against other proteins and sugars that cause agglutination of blood cells. Lectins, at low doses, interfere with nutrient digestion and decrease nutrient absorption. At high doses, they can trigger toxic effects and even death [209]. Interestingly, the activity of the lectin phytohemagglutinin (PHA) is lower in the raw faba bean (5.52 HU/mg) than in soy (692.82 HU/mg), beans (88.32 HU/mg), and lentils (11.01 HU/mg), but similar to peas (5.66 HU/mg) and higher than chickpeas (2.74 HU/mg) [29]. However, heating processes completely inhibit PHA activity, which makes faba bean consumption perfectly safe [29]. Partially purified and purified faba bean lectins were also shown to have antibacterial and antifungal activities [208]. Interestingly, the antifungal activities against *Candida albicans* were higher than pea and lentil activities. These results suggest that faba bean lectins could be used as natural antifungals.

### 4.3. Allergenicity

Faba bean proteins, as part of the legume family, can potentially induce hypersensitivity reactions. Thus, soybeans and peanutsare listed as priority allergens in North America [210] and lupin is part of the main allergens list in Europe [210]. Many allergens have been identified in other legumes, such as lentils, peas, and beans [211]. However, very little data are relatively available on faba bean allergens. A recent report compared the prevalence of sensitization to various legumes in a random group of allergic patients (*n* = 106) [212], and faba bean sensitization prevalence was among the lowest (5.7%), with green lentils (5.7%), when compared to black lentils (6.6%), white beans (7.5%), chickpeas (8.5%), soy (8.5%), blue lupine (8.5%), green peas (9.4%), white lupine (13.2%), and peanuts (14.2%).

A few reports published in the literature displayed clinical cases of allergic reactions to faba beans [213,214,215]. For instance, a woman in Italy experienced anaphylaxis shock after eating bread containing faba bean flour [213]. A 5-year-old boy experienced similar symptoms after eating a snack containing faba bean as well as other legumes and nuts [214]. An allergic reaction was also reported following faba bean handling by a farmer in Italy [215]. Allergenic specific responses to faba bean proteins were confirmed by a skin prick test (SPT) and specific IgE-binding protein identification in the three cases, suggesting that faba beans may contain numerous allergenic proteins and peptides.

Introducing a new dietary protein may induce allergenic reactions due to de novo sensitization or cross-reactivity with other allergens sharing structural similarities [216]. Faba bean protein cross-reactivity with other legumes and vegetables (fenugreek, red kidney bean, red gram, green gram, chickpea, and black gram) have been reported [217]. As the integration of new protein sources in the diet can provoke adverse health effects, it is essential to assess the allergenic potentials of novel protein sources before they are used in product formulations, to ensure public health safety [216]. Faba bean allergen risks deserve further assessments and characterizations, as with all new sources of proteins.

## 5. Conclusions

The faba bean has excellent nutritional and environmental advantages, its high protein content, combined with its agronomic features, makes it an ‘up-and-coming’ product that could be used to meet the shifting trends toward healthy eating and environmental consciousness. The faba bean has a balanced amino acid profile, which is complementary to cereal products, and its digestibility can be substantially increased through adequate processing strategies. Protein quality scores reported for faba beans remain lower than animal-based proteins, such as milk, eggs, and meat, which all have DIAAS value above 1.0 [218]. Nevertheless, with a diversified nutritious diet, amino acid requirements can be easily fulfilled from various plant sources [219]. Moreover, the classical definition of protein quality has been criticized because it does not take into account the whole food matrix composition that is known to have a significant influence on chronic disease preponderance and global health, which are major public health concerns [220]. Katz, Doughty, Geagan, Jenkins, and Gardner [220] recommended the introduction of health and environmental (based on life-cycle assessment) dimensions to the standard PDCAAS, with either a ratings system or adjustment factors to rank protein sources, on a basis that considers up-to-date scientific knowledge and health and environmental concerns. A reform of the protein quality definition would benefit plant-based protein sources and, more specifically, pulses, by acknowledging their environmental and health advantages. The faba bean can contribute to a healthy diet with its high content of dietary fibre, resistant starch, and minerals, among others. The faba bean is also a source of bioactive peptides and components that may procure noteworthy health benefits. However, further research is needed to better understand the health benefits and risks (particularly the allergenicity) associated with faba bean consumption to help increase and solidify its place in the growing and challenging global plant-based market.

## Figures and Tables

**Table 1:** Proximate compositions of the faba bean compared to pea and soy (g/100 g dry base) compiled from various studies [25,34,35,36,37,38,39,40,41,42,43,44,45,46,47,48,49,50,51,52,53,54,55,56,57,58,59,60,61,62,63,64,65,66,67].

Legume		Proteins		Carbohydrates					Ash	Fat
			TCH ^1^	Starch	Amylose ^2^	TDF ^3^	IDF ^4^	SDF ^5^		
Faba bean	Mean	27.6	66.0	40.0	34.0	12.9	15.1	1.4	3.4	1.4
	SD	3.0	5.1	3.4	6.4	9.0	4.6	1.8	0.4	0.4
	Min	22.7	55.2	28.1	18.6	6.4	10.7	0.6	2.6	0.7
	Max	34.7	71.4	47.5	44.4	34.9	30.3	7.6	4.4	3.2
	n1 ^6^	106	57	46	24	17	18	18	94	80
	n2 ^7^	13	6	7	3	6	4	4	11	11
Pea	Mean	23.4	63.5	44.9	29.6	14.7	11.0	2.5	3.0	1.6
	SD	2.4	7.1	1.2	3.5	2.6	0.9	1.4	0.3	0.5
	Min	18.1	52.8	42.2	19.1	12.2	9.7	1.7	2.4	1.0
	Max	27.5	70.0	46.6	31.6	19.4	12.9	5.6	3.7	2.9
	n1	34	5	18	12	11	8	8	23	23
	n2	12	4	6	3	6	3	3	10	10
Soy	Mean	40.0	28.6	2.7	-	21.9	24.8	2.6	5.2	19.7
	SD	3.0	3.0	2.7	-	8.3	8.6	2.3	0.6	2.2
	Min	31.5	19.7	0.2	-	13.7	15.4	0.6	3.0	14.0
	Max	46.8	33.2	6.7	-	35.5	32.6	6.1	6.3	23.6
	n1	48	31	19	-	9	5	5	40	60
	n2	12	5	2	-	5	4	4	8	12

^1^ TCH: total carbohydrate; ^2^ percentage of total starch; ^3^ TDF: total dietary fibres; ^4^ IDF: insoluble dietary fibres; ^5^ SDF: soluble dietary fibres; ^6^ n1: number of cultivars; ^7^ n2: number of references.

**Table 2:** Amino acid profiles of whole faba bean seeds compared to pea and soy.

	Faba Bean		Pea	Soy	Amino Acid Scoring Pattern ^1^
	cv. Bobas	cv. Kasztelan	cv. Solara	ND	
	(High tannin)	(Low tannin)			
Histidine *	2.41	2.29	2.52	2.91	2
Isoleucine *	3.94	3.91	3.33	4.6	3.2
Leucine *	7.47	7.01	6.58	7.76	6.6
Lysine *	7.08	6.71	6.84	7.08	5.7
Methionine *	0.87	1.06	1.03	1.29	2.7 ^2^
Cysteine	1.33	0.85	1.55	1.19	
Phenylalanine *	4.19	4.12	4.19	5.87	5.2 ^3^
Tyrosine	2.78	2.59	3.16	3.65	
Threonine *	3.40	3.40	3.59	3.69	3.1
Tryptophan *	0.87	0.85	0.94	1.38	0.85
Valine *	4.31	4.12	3.89	4.64	4.3
Arginine	9.46	9.04	6.84	8.86	.
Alanine	4.15	4.03	4.27	4.39	.
Aspartic acid	10.74	10.4	10.68	11.98	.
Glutamic acid	16.51	16.26	16.92	17.88	.
Glycine	4.73	4.25	4.32	4.20	.
Proline	3.94	3.86	3.76	4.92	.
Serine	4.69	4.76	4.79	4.77	.
References	[105]	[105]	[108]	[105]	[100]

* Essential amino acids. ^1^ Amino acid scoring pattern for children 6 months to 3 years, recommended for regulatory purposes by the FAO [100]. ^2^ Sulfur-containing amino acids (methionine and cysteine). ^3^ Aromatic amino acids (phenylalanine and tyrosine).

**Table 3:** Bioactive peptides isolated from faba bean protein hydrolysates.

	Amino Acids Sequence	Bioactive Properties	Starting Material	Protein Hydrolysis Method	Protein Precursor	Hydrophobic Residue (%)	References
1	GGQHQQEEESEEQK	Antioxidant (DPPH assay) Antibiofilm (biofilm inhibition of *Pseudomonas aeruginosa PA14*)	Faba bean protein isolate	Trypsin hydrolysis (18 h 37 °C)	Legumin	0	[74]
2	GPLVHPQSQSQSN	Antioxidant (DPPH assay) Antityrosinase (tyrosinase inhibition assay)			Legumin	15	
3	LSPGDVLVIPAGYPVAIK	Antioxidant (DPPH, FRAP, and ferrous ion-chelating assays) Antibiofilm (biofilm inhibition of *Pseudomonas aeruginosa PA14*)			Vicilin	56	
4	VESEAGLTETWNPNHPELR	Antioxidant (DPPH assay), Antityrosinase (tyrosinase inhibition assay) Antibiofilm (biofilm inhibition of *Pseudomonas aeruginosa PA14*)			Legumin	26	
5	EEYDEEKEQGEEEIR	Antioxidant (DPPH assay) Antibiofilm (biofilm inhibition of *Pseudomonas aeruginosa PA14*)			Vicilin	13	
6	ELAFPGSAQEVDTLLENQK	Fungicide	Lentil, pea and faba bean flours mixed (1:1:1)	Veron^®^ PS (6 h 30 °C)	Vicilin	36	[142]
7	LSPGDVLVIPAGYPVAIK	Fungicide			Vicilin	50	
8	SAQ	Promoting lactic acid bacteria growth	Faba bean protein isolate	Alcalase (1 h 37 °C)	ND	ND	[143]
9	Peptide enriched fraction						
	DALEPDNRIESEGGLIETWNPNNRQ	Antioxidant (ABTS assay) Antihypertensive (ACE inhibition) Anti-inflammatory (LOX inhibition) Reduction of lipids absorption (pancreatic lipase inhibition)	Fermented faba bean flour	In vitro simulated gastrointestinal digestion	Legumin	ND	[73]
	FEEPQQSEQGEGR						
	GSRQEEDEDEDE						
	WMNYNDQIPVINNQLDQMPR						
	RGEDEDDKEKRHSQKGES						
	RLNIGSSSSDIYNPQAGR						
10	HLPSYSPSPQ	Promote muscle protein synthesis (increased phosphorylation S6 in skeletal muscle cells)	Faba bean protein powder	Food grade endopeptidase	ND	ND	[144]
11	TIKIPAGT	Anti-inflammatory (Reduced TNF-α in macrophages)					

## Data Availability

Not applicable.
